# Evaluation of faba beans as an ingredient in dog diets: apparent total tract digestibility of extruded diets with graded levels of dehulled faba beans (*Vicia faba* L.) by dogs

**DOI:** 10.1093/jas/skaa085

**Published:** 2020-03-24

**Authors:** Isabella Corsato Alvarenga, Dalton Holt, Charles G Aldrich

**Affiliations:** Department of Grain Science and Industry, Kansas State University, Manhattan, KS

**Keywords:** blood parameters, canine nutrition, digestibility, legumes, palatability, trypsin inhibitor

## Abstract

The growing pet food market is continuously in search for novel ingredients. Legumes such as faba beans (**FB**) are increasing in popularity and are known to benefit human health, but little is known about their use in pet foods. The objective of this study was to determine the effect of dehulled FB utilization by dogs. Experimental diets were extruded with 0%, 10%, 20%, and 30% FB inclusion (FB0, FB10, FB20, and FB30, respectively). Beagle dogs (*n* = 12) were fed the diets for 9-d adaptation with 5-d total fecal collection in a replicated 4 × 4 Latin square design. Apparent total tract digestibility (**ATTD**) was determined by external marker Cr_2_O_3_. At the culmination of each period, blood samples were collected from brachial venipuncture for complete blood count and blood chemistry. Palatability was determined with a 2-bowl test (*n* = 20). Means of blood parameters were separated by multivariate analysis of variance (MANOVA) with the aid of statistical software (SAS v9.4). Contrasts and least square means of fecal parameters and ATTD were computed. Significance level was considered to be α = 0.05. Dogs ate all food on offer and maintained body weight. There was no difference (*P* > 0.05) among treatments (FB10, FB20, and FB30) and the control (FB0) relative to food intake, fecal output (“as is” basis), and fecal score, but feces were softer when dogs were fed the treatments (*P* = 0.031) and there was a linear increase (*P* = 0.011) in defecation frequency (stools/day) when FB increased in the diets. Dry matter, organic matter, and crude protein digestibilities were slightly higher when dogs were fed the control diet (*P* < 0.05) compared with the FB diets. All blood and serum chemistry parameters were similar among treatments and within the reference ranges. Dogs preferred the control diet relative to the 10% and 30% FB diets, but the 20% FB preference was similar to the control. Dogs remained healthy, maintained body weight and no adverse health events were observed during the study. Dehulled FB are a suitable ingredient for dog foods, but concentrations should not exceed 20% to avoid reduction in palatability and stool quality.

## Introduction

In the pet food market, sales are mostly driven by premium foods with claims for health, natural, organic, fortified, functional, weight control, life stage, and (or) gourmet. In this continually fragmenting market, novel ingredients have been at the forefront of new product entrants. Many ingredients, such as peas, chickpeas, potatoes, and tapioca, have been used in pet foods for years with few issues. Whereas, less common ingredients, such as faba beans (**FB**; *Vicia faba*), have recently started to be explored. FB, also known as broad beans, field bean, bell bean, or tic bean, are native to North Africa and West Asia, with the largest production in China (Akibode and Maredia, 2012). FB are a good protein and starch source, and if processed properly can be an effective ingredient in monogastric diets. Pulses like faba are also rich in folate (Hoppner and Lampi, 1993) and they provide a wide range of benefits to human health (Campos-Vega et al., 2010). Some important health benefits attributed to phytochemicals from pulses include antioxidative potential and anticancer activity (Rochfort and Panozzo, 2007).

FB consumption can lead to some digestive issues if not properly cooked. This is due to antinutritional factors in the beans which are inactivated during thermal processing (Alonso et al., 2000). Further, some people are susceptible to favism, a blood disorder caused by condensed tannins in the seed hull of FB under certain conditions (Crépon et al., 2010). Also, the presence of oligosaccharides in FB that escape digestion and are fermented in the colon have been associated with flatulence in humans (Rochfort and Panozzo, 2007). This information is not available for dogs. Thus, the objective of this work was to determine apparent total tract digestibility (**ATTD**), stool quality, and hematology of dogs fed extruded kibbles containing incremental levels of dehulled FB.

## Materials and Methods

### Ingredients and diets

Four nutritionally complete experimental diets were formulated, in which rice and corn gluten meal were replaced by dehulled FB at 10%, 20%, and 30% of the formula (Table 1). Ground FB was provided by the study sponsor (3D Corporate Solutions; Monett, MO, USA), and brewers rice, beet pulp, and corn gluten meal were purchased from a local mill (Fairview Mills, LLC, Seneca, KS, USA). Diets were formulated to have similar macro- and micronutrient composition and included chromic oxide (Cr_2_O_3_) and titanium dioxide (TiO_2_) to serve as external markers. Only data from Cr_2_O_3_ are presented in this manuscript. Mixing and extrusion were conducted at a local extrusion pilot plant (Extrutech Inc.; Sabetha, KS, USA) under procedures fully described previously by Corsato Alvarenga and Aldrich (2019). Extruded foods and ground FB were tested for trypsin inhibitor units (**TIU**; AOCS Ba 12–75) at a commercial laboratory (Eurofins Scientific, Des Moines, IA, USA).

**Table 1.** Ingredient composition of diets with increasing levels of FB (FB0, 0%; FB10, 10%; FB20, 20%; and FB30, 30%)

Ingredient, %	Control	FB10	FB20	FB30
FB, dehulled	0.00	10.00	20.00	30.00
Rice, brewers	44.59	37.90	32.00	26.10
Chicken meal, low ash	31.85	28.98	28.91	28.84
Corn gluten meal, 60%	10.00	9.14	5.20	1.25
Chicken fat (topical)	6.25	6.61	6.58	6.56
Beet pulp	4.00	4.00	4.00	4.00
Digest—dry dog flavor	1.00	1.00	1.00	1.00
Salt	0.650	0.650	0.650	0.650
Potassium chloride	0.325	0.250	0.250	0.250
Choline chloride, 60% dry	0.200	0.200	0.200	0.200
Dicalcium phosphate	0.033	0.171	0.108	0.045
Fish oil	0.145	0.145	0.144	0.144
Dry natural antioxidant^1^	0.034	0.033	0.033	0.033
Liquid natural antioxidant^1^	0.031	0.033	0.033	0.033
Vitamin premix^2^	0.150	0.150	0.150	0.150
Trace mineral premix^3^	0.100	0.100	0.100	0.100
Chromium sesquioxide	0.250	0.250	0.250	0.250
Titanium dioxide	0.400	0.400	0.400	0.400

^1^Dry and liquid natural antioxidant: mixed tocopherols, citric acid, rosemary extract, and soybean oil.

^2^Vitamin premix: 5.51% moisture, 4.02% crude protein, 34.5% ash, 13.4% calcium, 17,162,999 IU/kg Vitamin A, 920,000 IU/kg Vitamin D, 79,887 IU/kg Vitamin E, 14,252 mg/kgthiamine, 4,719 mg/kg riboflavin, 12,186 mg/kg pantothenic acid, 64,736 mg/kg Niacin, 5,537 mg/kg pyridoxine, 720 mg/kg Folic acid, 70 mg/kg biotin, 22 mg/kg vitamin B12.

^3^Trace mineral premix: 0.66% moisture, 21.5% calcium, 0.02% sodium, 0.57% magnesium, 38,910 mg/kg iron, 11,234 mg/kg copper, 5,842 mg/kg manganese, 88,000 mg/kg zinc, 1,584 mg/kg iodine, 310 mg/kg selenium, 19% carbohydrate, and 1% crude fat.

### Animals and digestibility estimations

The digestibility experiment was approved by the Kansas State University Institutional Animal Care and Use Committee (IACUC) under protocol #3730. Twelve intact adult Beagle dogs (eight male and four female) of similar age (range 2.2 to 2.8 yr) and initial body weight (10.71 kg ± 0.885; mean ± SD) were housed in a climate-controlled building with temperature between 22 and 32 °C at the Large Animal Research Center at Kansas State University (Manhattan, KS, USA). Dogs were kept in individual cages (1.83 × 1.20 m) with an acrylic-mesh floor and three-piece pans underneath. Each group of three animals was randomly assigned to a specific diet (**FB0**, rice-based diet with no FB; **FB10**, diet with 10% FB; **FB20**, diet with 20% FB; **FB30**, diet with 30% FB) for a period of 9-d adaption, followed by 5 d fecal collection in a replicated 4 × 4 Latin square design (Kim and Stein, 2009). Thus, there was a total of 12 observations per treatment and each dog served as their own control. All dogs began the study with a body condition score ranging between 4 and 6 on a 9-point scale (Laflamme, 1997).

As a starting point to determine the dietary intake, dogs’ daily metabolizable energy (**ME**) requirements were calculated as an average for laboratory kennel dogs or active pet dogs: 132 * BW^0.75^ (NRC, 2006). The ME of finished diets were calculated using the predicted equations in dog foods (NRC, 2006). Food offered (grams) was a result of the daily ME relative to the predicted food ME. Dogs were weighed biweekly and their food allowance adjusted by 5% or 10% when necessary. Dogs had free access to water at all times and food was split into two meals per day (at 0800 and 1700 hours). Feces were assessed for consistency using a 5-point scale with 0.5 point increments; where 1 = liquid diarrhea, 2 = very soft paste-like, does not retain shape; 3 = soft feces, retains shape; 4 = firm formed feces (ideal score), and 5 = very hard, dry pellets. Total fecal output was calculated using external marker chromic oxide as described by Alvarenga et al. (2019). At the end of each feeding period, approximately 7 mL blood from each dog was collected from the brachial vein and divided into two test tubes: one with ethylenediaminetetraacetic acid (**EDTA**; BD Vacutainer plastic blood collection tubes with K_2_EDTA) to be analyzed for complete blood count (**CBC**), and the second without EDTA (BD Vacutainer venous blood collection tubes: SST serum separation tubes) to be analyzed for blood chemistry. Tubes were placed in ice immediately after collection and analyzed on the same day at the Kansas State Veterinary Diagnostic Laboratory (Manhattan, KS, USA).

At the culmination of the feeding study, wet fecal samples were weighed, then dried at 55 °C until the moisture level was below 9%. This initial drying step avoided bacterial or mold growth until fecal nutrients were analyzed. After this step, both fecal and food samples were ground in a laboratory fixed blade impact mill (Retsch, type ZM200; Haan, Germany) to pass a 1-mm screen. Samples were stored in glass jars at room temperature until further analysis.

Chromium was determined in both food and feces according to the procedure by Williams et al. (1962). ATTD was calculated according to equation I:

$$\begin{array}{l} Nutrient\;ATTD=\frac{\left[1-\left(\%\;Cr\;in\;food\;\times \%\;nutrient\;in\;feces\right)\right]\times 100}{\left(\;\%\;Cr\;in\;feces\times \%\;nutrient\;in\;food\right)} \end{array}$$ (I)

### Blood parameters

Complete blood count was analyzed by a high-volume hematology analyzer (Siemens Advia 2120i; Siemens Healthcare GmbH; Erlangen, Germany). The parameters measured were leukocyte count, erythrocyte concentration, hemoglobin, cellular hemoglobin, hematocrit, mean cell volume, mean cell hemoglobin, mean cell hemoglobin concentration, cell hemoglobin concentration mean, cellular hemoglobin content, red blood cell (**RBC**) distribution width, mean platelet volume, absolute reticulocyte, reticulocyte cellular hemoglobin, reticulocyte mean cell volume, segmented neutrophil concentration, band neutrophil concentration, lymphocyte concentration, monocyte concentration, eosinophil concentration, basophil concentration, hematocrit (spun), and plasma protein. Blood chemistry was performed by a mid-volume analyzer (Roche Cobas C501; Roche Diagnostics, Indianapolis, IN, USA). Parameters analyzed were glucose, urea nitrogen, creatinine, total protein, albumin, globulin, calcium, phosphorus, sodium, potassium, chloride, bicarbonate, anion gap, sodium/potassium, alanine transaminase P5P, alkaline phosphate, creatine kinase, and cholesterol. Reference intervals for serum chemistry and CBC parameters were established by the Kansas State Veterinary Diagnostic Laboratory following the guidelines provided by the American Society for Veterinary Clinical Pathology (ASVCP; Friedrichs et al., 2012) and in compliance with the American Association Veterinary Laboratory Diagnosticians (AAVLD) accreditation requirements.

### Nutrient analyses

Experimental foods and feces were analyzed for moisture and dry matter (Association of Official Analytical Chemists (**AOAC**) 930.15), ash (AOAC 942.05; organic matter calculated as dry matter % − ash %), crude protein (AOAC 990.03), crude fat by acid hydrolysis (AOAC 954.02), and minerals (AOAC 985.01 modified) at a commercial analytical laboratory (Midwest Laboratories, Omaha, NE, USA). Total dietary fiber (**TDF**) was measured in both diets and feces following the standard procedure from the Total Dietary Fiber Analysis Kit (TDF100A, Sigma-Aldrich; Saint Louis, MO). Insoluble and soluble dietary fiber fractions were also measured in diets using a modified procedure from the standard kit. Briefly, insoluble fiber was determined following the protocol without the addition of 190 proof ethanol after enzymatic digestion. Soluble fiber was calculated by the difference between total and insoluble fiber. Gross energy was determined in an adiabatic bomb calorimeter (bomb calorimeter model 1351, Paar Instruments; Moline, IL, USA). Finally, essential amino acid composition of the diets were calculated prior to the conduct of the experiment from a commercial formulation database, whereas essential amino acid composition of the FB were measured according to AOAC (994.12; Midwest Laboratories, Omaha, NE) at the conclusion of the study.

### Palatability assessment

Palatability testing was conducted at a commercial research kennel (Summit Ridge Farms; Susquehanna, PA, USA) as a two-pan forced-choice test. Twenty Beagle dogs were presented with a choice between two products (400 g each) in stainless steel bowls and selection of the first choice was recorded, along with total quantity of food consumed from both bowls during 30 min. In these experiments, all treatments were compared with the control (three palatability tests), and each comparison was carried out for 2 d, switching the bowls on the second day to avoid side bias.

### Statistical analysis

Fecal samples were collected per day of collection (total 5 d per period), then combined after drying, and nutrient ATTD data were determined per dog in each period. This was one observational unit. Dependent variables included food intake, fecal scores, number of defecations per day, wet and dry fecal output, and nutrient ATTD, while dog, period, and diet were the fixed or independent variables. Parametric data were analyzed as a replicated 4 × 4 Latin square design using general linear models procedure of SAS (v. 9.4; SAS Inst. Inc., Cary, NC). Fecal scores (nonparametric) were analyzed as repeated measures using the generalized linear mixed models procedure of SAS. Treatment means were compared by single degree of freedom contrasts and *P*-values were reported for “control vs. treatments,” linear, quadratic, and cubic effects of fecal parameters and nutrient ATTD by dogs fed each treatment, with a significance level of α = 0.05. Blood parameters means were separated by multivariate analysis of variance (MANOVA) and grouped using the Tukey correction method at a significance of *P* < 0.05. A two-way analysis of variance (**ANOVA**) was used to compare the consumption difference between the two diets (control vs. treatment) in the palatability study and were considered significant at a *P* < 0.05. First choice daily counts were compared between the control and treatments using the Chi-square test and were considered significant at a *P* < 0.05.

## Results

The diets were slightly drier than the target (<6% moisture) but within normal production parameters (Table 2). The crude protein content was similar among diets (average 37.1%). Crude fat had a 2.1 percent unit difference between FB20 and FB30 diets, which could be due to sampling error or analytical variation. TDF tended to increase as FB was added to the diets, and insoluble fiber represented over 75% of the TDF content in both FB and diets. Ash content of samples was close to 5% as expected. The concentration of minerals calcium, phosphorous, potassium, magnesium, sodium, sulfur, manganese, copper iron, and zinc were similar among diets and met AAFCO (2019) and NRC (2006) recommendations for adult dogs at maintenance (Table 2). Amino acids were calculated on the formulas prior to the experiment and were targeted to meet the nutritional requirements for maintenance ( NRC, 2006; AAFCO, 2019). Dehulled FB amino acid profile was measured following the study and appears to be similar to other legumes; wherein, methionine and cystine were likely the first limiting (Donadelli et al., 2019). The addition of other ingredients such as corn gluten meal and chicken meal would compensate for these limitations in the present study.

**Table 2.** Nutrient analysis on a dry matter basis of raw dehulled FB and post-extrusion experimental diets with increasing levels of dehulled FB (FB0, 0%; FB10, 10%; FB20, 20%; and FB30, 30%)

Item	FB	FB0^1^	FB10^1^	FB20^1^	FB30^1^
Dry matter, %	87.4	94.3	95.7	94.1	96.5
Crude protein, %	32.0	36.3	36.6	37.5	38.1
Crude fat, %	1.57	13.4	12.5	14.2	12.1
TDF, %	8.11	3.12	4.32	4.39	5.08
Insoluble fiber, %	6.31	3.02	3.68	3.37	2.29
Soluble fiber, %	1.803	0.107	0.643	1.016	2.791
Ash, %	3.51	5.54	5.53	5.60	6.02
Calcium, %	0.07	0.91	0.91	0.91	0.88
Phosphorous, %	0.55	0.76	0.80	0.73	0.77
Potassium, %	1.30	0.63	0.74	0.59	0.80
Magnesium, %	0.14	0.09	0.10	0.08	0.1
Sodium, %	n.d.^2^	0.47	0.47	0.48	0.44
Sulfur, %	0.19	0.44	0.42	0.46	0.37
Manganese, ppm	19.4	21.3	21.9	21.1	18.3
Copper, ppm	14.1	18.2	18.9	17.7	19.1
Iron, ppm	67.3	124	128	127	117
Zinc, ppm	56.5	140	135	130	138
Arginine	2.86	1.86	1.92	2.10	2.20
Histidine	0.77	0.56	0.57	0.60	0.60
Isoleucine	1.43	1.11	1.12	1.14	1.13
Leucine	2.43	2.99	2.88	2.68	2.36
Lysine	1.99	1.53	1.56	1.72	1.81
Methionine	0.21	0.61	0.57	0.55	0.51
Phenylalanine	1.44	1.29	1.27	1.24	1.16
Threonine	1.17	1.05	1.04	1.07	1.05
Tryptophan	0.38	0.23	0.23	0.23	0.23
Valine	1.38	1.34	1.33	1.35	1.32

^1^Amino acids of all diets (FB0, FB10, FB20, and FB30) were calculated prior to the experiment.

^2^n.d., not detected.

There was a small amount of variation in weight gain or loss of the dogs from the beginning to the end of the study (range between 4.27% loss and 5.15% gain), but weight change among treatments within each period was not significant (*P* > 0.05) according to a one-way ANOVA test performed using a statistical software (JMP Pro 14.2.0, Cary, NC). The assay for TIU has a limit of quantification (**LOQ**) of 1,000 TIU/g. Results for FB0, FB10, FB20, and FB30 were similar and close to the LOQ, and 3 × lower than that of ground FB ingredient (1,200 TIU/g, 1,200 TIU/g, 1,100 TIU/g, <1,000 TIU/g, and 3,600 TIU/g for FB0, FB10, FB20, FB30, and FB, respectively).

All food was readily consumed by all dogs throughout the study, with the exception of 2 d in which minor amounts of orts were measured. These were subtracted from the total intake. Food intake, fecal output (dry matter [**DM**] and as-is, determined by chromic oxide marker), and defecation frequency were not different among the control (FB0) and treatments (*P* > 0.05), but there was a linear incline (*P* < 0.05) in fecal output and defecation frequency as FB level increased in the diets (Table 3). Stool scores for dogs fed the control diet were on average higher (*P* = 0.031) than those fed the FB treatments, which would be expected when increasing soluble fiber in the diet.

**Table 3.** Least square means and contrasts (FB0 vs. FB10–30 [*T*]; linear [*L*]; quadratic [*Q*]; cubic [*C*]) for food intake, fecal output, stool scores, and defecation frequency of dogs fed diets containing increasing levels of dehulled FB

Parameter	FB0	FB10	FB20	FB30	SEM	FB0 vs. *T*	*L*	*Q*	*C*
Intake (DM), g/d	147	148	152	147	6.1	0.735	0.847	0.631	0.732
Fecal output (DM), g/d	17.4	18.1	17.9	19.1	0.54	0.108	0.045	0.714	0.331
Fecal output (as-is), g/d	56.6	62.2	61.5	70.6	4.78	0.147	0.061	0.721	0.461
Stool score^1^	3.77	3.65	3.72	3.53	*	0.031	0.006	0.469	0.051
Defecation frequency, stools/d	1.52	1.62	1.73	1.85	0.093	0.052	0.011	0.947	0.960

^1^Subjective 1 to 5 scale with 1, runny; 2, soft; 3, firm and moist; 4, firm; 5, dry and hard.

*SEM differed for each treatment due to unequal number of observations (SEM; 0.051, 0.049, 0.052, 0.043, for FB0, FB10, FB20, and FB30, respectively). Animal (*P* = 0.001) and diet (*P* = 0.007) had a significant effect on fecal scores, but there was no effect on the interaction.

Nutrient digestibility had small variations among treatments. Dry matter and organic matter ATTD had a slight decline in a linear fashion as FB increased in the diets (*P* < 0.001; Table 4). Gross energy ATTD for control vs. the FB treatments were not different, but there was a cubic relationship due to an inconsistent effect of the FB20 diet. Crude protein ATTD was slightly higher for dogs fed the control vs. FB treatments (FB0 vs. T; *P* < 0.05). Fat ATTD was high and similar across treatments (average 94.2%). TDF ATTD was lower for dogs fed the control (FB0 vs. T; *P* < 0.001) and there was a linear increase as FB content increased (*P* < 0.001). Finally, ash ATTD was not different for FB treatments compared with the control, but a quadratic relationship (*P* < 0.05) was observed among treatments.

**Table 4.** Least square means and contrasts (FB0 vs. FB10–30 [*T*]; linear [*L*]; quadratic [*Q*]; cubic [*C*]) for nutrient ATTD by dogs fed diets with increasing levels (0%, 10%, 20%, and 30%) of dehulled FB

ATTD	FB0	FB10	FB20	FB30	SEM	FB0 vs. *T*	*L*	*Q*	*C*
Dry matter, %	89.1	88.4	88.8	88.0	0.14	<0.001	<0.001	0.514	0.001
Organic matter, %	92.7	91.7	92.2	91.4	0.14	<.0001	<0.001	0.713	0.001
Gross energy, %	83.7	83.6	84.2	82.6	0.27	0.475	0.032	0.010	0.035
Crude protein, %	90.8	89.6	90.7	90.0	0.21	0.004	0.107	0.250	0.001
Fat, %	94.4	94.0	94.5	93.9	0.14	0.156	0.239	0.539	0.002
TDF, %	1.36	17.64	20.72	21.98	2.330	<0.001	<0.001	0.002	0.280
Ash, %	34.6	31.8	35.3	36.6	0.56	0.925	0.001	0.001	0.0014

All dogs remained healthy during the study which was confirmed by blood composition. The CBC of dogs were not different for any parameter among treatments and were within normal reference ranges (Table 5). Likewise, serum chemistry was also unaffected by treatment and all parameters were within the reference range (Table 6).

**Table 5.** Complete blood count results for dogs fed diets containing increasing levels of dehulled FB (0%, FB0; 10%, FB10; 20%, FB20; and 30%; FB30)

Parameter	FB0	FB10	FB20	FB30	SEM	*P*-value	Reference^1^ range
Leukocyte count, K/uL	7.28	7.26	7.20	7.47	0.22	0.846	4.3 to 13.6
Erythrocyte concentration, M/uL	7.49	7.43	7.57	7.48	0.18	0.960	5.8 to 8.20
Hemoglobin, g/dL	17.98	17.81	18.14	17.84	0.39	0.932	14.1 to 20.5
Cellular Hemoglobin, g/dL	18.22	18.07	18.33	18.12	0.44	0.974	14.1 to 20.6
Hematocrit, %	52.81	52.49	53.17	52.56	1.25	0.981	41.0 to 59.0
Mean cell volume, fL	70.62	70.68	70.33	70.47	0.63	0.980	64.0 to 76.0
Mean cell hemoglobin, pg	24.03	24.01	23.99	23.93	0.18	0.983	22.0 to 26.0
Mean cell hemoglobin concentration, g/dL	34.05	33.97	34.13	33.98	0.20	0.933	33.0 to 36.0
Cell hemoglobin concentration. Mean, g/dL	34.48	34.43	34.5	34.48	0.11	0.967	33.0 to 36.0
Cellular hemoglobin content, pg	24.26	24.23	24.18	24.22	0.22	0.996	No Ref.
RBC distribution width, %	12.83	12.82	12.68	12.67	0.14	0.731	11.4 to 13.7
Mean platelet volume, fL	10.67	10.77	10.76	10.71	0.18	0.977	8.3 to 15.3
Absolute reticulocyte, M/uL	0.037	0.039	0.038	0.037	0.006	0.989	0.01 to 0.12
Reticulocyte cellular hemoglobin, pg	25.73	25.74	25.78	25.77	0.21	0.998	23–28
Reticulocyte mean cell volume, fL	93.07	94.36	94.67	94.22	0.84^1^	0.568	78 to 100
Segmented neutrophil concentration, K/uL	4.46	4.38	4.59	4.66	0.18	0.680	2.5 to 9.3
Band neutrophil concentration, K/uL	0.017	0.017	0.018	0.008	0.012^1^	0.914	0.0 to 0.1
Lymphocyte concentration, K/uL	2.22	2.19	1.99	2.08	0.14^1^	0.653	0.8 to 4.3
Monocyte concentration, K/uL	0.29	0.43	0.35	0.40	0.06^1^	0.393	0.1 to 0.9
Eosinophil concentration, K/uL	0.29	0.25	0.25	0.28	0.05^1^	0.920	0.0 to 1.5
Basophil concentration, K/uL	0.008	0.000	0.000	0.008	0.005^1^	0.595	0.0 to 0.1
Hematocrit (spun), %	51.17	51.00	52.18	50.75	1.16^1^	0.842	40 to 57
Plasma protein, g/dL	6.94	6.90	7.01	6.97	0.11^1^	0.919	6.3 to 8.0

^1^ Friedrichs et al. (2012).

**Table 6.** Blood chemistry profiles for dogs fed diets containing increasing levels of dehulled FB

Parameter	FB0	FB10	FB20	FB30	SEM	*P*-value	Reference^1^ range
Glucose, mg/dL	87.25	88.25	92.64	86.58	3.30	0.593	70 to 120
Urea nitrogen, mg/dL	17.00	16.83	16.27	16.30	0.53	0.708	8 to 29
Creatinine, mg/dL	0.73	0.72	0.73	0.73	0.04	0.990	0.6 to 1.4
Total protein, g/dL	6.42	6.41	6.47	6.38	0.11	0.951	5.3 to 6.9
Albumin, g/dL	3.75	3.69	3.70	3.71	0.07	0.920	3.2 to 4.2
Globulin, g/dL	2.67	2.72	2.77	2.68	0.08	0.818	1.8 to 3.0
Calcium, mg/dL	10.53	10.41	10.47	10.44	0.10	0.869	9.5 to 11.2
Phosphorus, mg/dL	3.89	4.01	4.00	3.95	0.12	0.896	2.2 to 6.1
Sodium, mmol/L	148.67	148.75	149.36	149.17	0.53	0.764	144 to 151
Potassium, mmol/L	4.78	4.73	4.76	4.72	0.05	0.789	3.7 to 5.0
Chloride, mmol/L	111.42	111.67	112.27	111.83	0.55	0.751	106 to 117
Bicarbonate, mmol/L	19.06	18.10	18.74	19.09	0.53	0.524	18 to 24
Anion gap, mmol/L	23.92	24.75	24.18	24.09	0.78	0.888	18 to 27
Sodium/potassium	31.08	31.50	31.36	31.58	0.40	0.822	30 to 39
Alanine transaminase P5P, U/L	41.25	38.92	39.36	41.75	3.82	0.941	20 to 144
Alkaline phosphate, U/L	36.00	36.92	63.18	33.75	12.31	0.325	10 to 130
Creatine kinase,U/L	168.17	174.00	162.18	205.75	17.99	0.341	54 to 226
Cholesterol, mg/dL	193.75	188.33	171.91	187.5	19.66	0.885	140 to 390

^1^ Friedrichs et al. (2012).

Results from the two-bowl palatability tests indicated that the control was preferred by the dogs over the FB10 and FB30, with 32 and 33 first choices (out of 40) and intake ratio of 0.752 and 0.855 (Table 7). Interestingly, when dogs were offered the FB20 diet, first-choice preference and intake ratio were similar (*P* > 0.05) to the control.

**Table 7.** Palatability assessment of diets containing dehulled FB relative to the control (0% dehulled FB) by dogs

Diet A vs. B	FC, *n*^1^	IR of diet A^2^
FB0 vs. FB10	32*	0.752*
FB0 vs. FB20	22	0.439
FB0 vs. FB30^3^	33*	0.855*

^1^FC (first choice) number of first visits to bowl with diet B can be obtained by 40-*n*.

^2^IR (intake ratio) of diet A = average of intake (g) of diet A/total intake (g) of diets A + B.

^3^One dog did not make a choice on the first day.

*P-value is less than 0.05.

## Discussion

This study is to our knowledge the first to explore FB as an ingredient in dog foods. All diets were well utilized by dogs, with no overt health issues observed and no impact on blood cell count or serum biochemistry profile of dogs. Based on prior experience of the authors, 30% FB inclusion was considered to be the limit for legume seeds in a dog food formula so that gastrointestinal intolerance would not be an issue. More typical levels would be 10% to 20%. Given the novelty of these beans to dogs and issues that have been reported in humans, there was a need to explore the upper levels of inclusion to detect any adverse effects from FB.

From the work of Corsato Alvarenga and Aldrich (2019), the oligosaccharide levels in FB were 33.5 mg/g sucrose, 2.62 mg/g raffinose, 6.96 mg/g stachyose, and 25.3 mg/g verbascose, which are highly fermentable components of the diet soluble fiber fraction. The amount of TDF in the FB was much lower than what is commonly reported in whole legumes (Bednar, 2001). This was expected because the FB were dehulled, and the hull contains most of the insoluble fiber. The soluble fiber fraction of the FB was comparable to other legumes (1.8% vs. average 1.3%; Bednar et al., 2001). Sunvold et al. (1995) reported that dogs fed diets with more fermentable fiber sources such as beet pulp and citrus pulp had a higher wet fecal output and more loose stools. Oligosaccharides are also known to cause a laxative effect (Flickinger et al., 2000). This is because these carbohydrates are indigestible by small intestinal enzymes and become a substrate for bacterial fermentation in the colon, which in excess can shift the osmotic balance and lead to an increase in fecal moisture. This is in part why legume seeds are often limited in pet foods. As expected, at higher FB levels, there was a tendency to increase the number of defecations per day, and stools tended to soften. Moreover, extra precaution should be taken when feeding high legume diets to large dogs, as they are more susceptible to forming looser stools than smaller breeds (Goudez et al., 2011).

In the present study, brewers rice and corn gluten meal were replaced proportionally by FB in order to maintain the same concentration of crude protein across treatments. There was a small decrease (close to 1%) in the overall ATTD when FB was added at increasing levels, but not to physiologically meaningful levels for the animal. Cargo-Froom et al. (2019) also explored a high percentage of legumes (12% chickpeas and lentils and 19% green and yellow peas) in dog diets vs. an animal-based diet and reported that the vegetarian food led to improved organic matter (**OM**) ATTD. Although their approach was different from the present study, they reported legumes to be well utilized by dogs. Forster et al. (2012) also reported that a diet with 25% navy beans was well utilized by dogs in comparison to their control diet, but both diets had a DM and OM ATTD much lower than what was found in the present study, and what is usual for pet foods in the literature (Carciofi et al., 2008; Cargo-Froom et al., 2019). Since this happened in both treatments, the low ATTD rates in Forster’s work were likely an effect of the study design rather than the navy beans directly. In their study, dogs were from various breeds and feces were collected by the owners, which could have led to higher variation in ATTD and incomplete collections.

There may be concerns regarding FB consumption by dogs relative to blood disorders such as favism or poor digestibility caused by antinutritional factors. Favism only develops in humans with glucose-6-phosphate dehydrogenase (**G6PD**) deficiency and is triggered by the β-glucosides vicine and convicine, which are present in FB (Luzzatto and Arese, 2018). The outcome is acute hemolytic anemia, with hemoglobinuria and moderate to severe signs of anemia (Luzzatto, 1973). Generally, β-glucosides are largely inactivated when cooked (Arese and De Flora, 1990), and favism only occurs when there are both a G6PD deficiency and large consumption of FB (Luzzatto and Arese, 2018). Thus, the risk of dogs developing favism was assumed to be extremely low before conducting the study, specifically because biogenic amines were very low or nonexistent in the diets after extrusion (Corsato Alvarenga and Aldrich, 2019). Within the study, there were no signs of anemia or abnormal hematology, although a longer feeding period would be required to confirm this. Forster et al. (2012) measured blood parameters of dogs after eating cooked navy beans and reported results within reference ranges as well, so the authors concluded that cooked navy beans, which are pulses like FB, were tolerated. A 2-wk feeding period does not provide enough time to draw conclusions regarding long-term health. To do that, a longer feeding time such as the 26 wk required to substantiate an adult maintenance claim for a dog food (AAFCO, 2019) would be more telling. However, there was an indication that after inclusion in an extruded food beans were safe for consumption (Alonso et al., 2000; Corsato Alvarenga and Aldrich, 2019).

It has been demonstrated in the past that factors such as hemagglutinating activity, trypsin and chymotrypsin inhibitors in peas are deactivated during extrusion (Alonso et al., 2000). Trypsin inhibitor activity of all diets in the present study was close to the LOQ, so these could essentially be considered below a level that would cause an effect. Biogenic amines such as histamine, putrescine, cadaverine were measured in the dehulled FB and FB0, FB10, FB20, and FB30 diets and were reported previously (Corsato Alvarenga and Aldrich, 2019). These also were close to 0% in the diets after extrusion. Thus, there were sufficient data to support the use of thermally processed dehulled FB in dog foods, even though the study was short, since all the animals were healthy before and after consumption of each treatment.

Besides nutrient digestibility, palatability is also an important factor that pet food companies consider when making a dog or cat food. Although not as palatable when compared with the control at 10% and 30 % FB, the intake ratio at 20% would suggest that preference might be modified to make the foods containing FB acceptable to the animal. What factors led to this inconsistency were not immediately apparent. However, when fed in the digestibility study, dogs ate all food offered readily.

## Conclusion

All dogs remained healthy during the study, which was confirmed by CBC and blood chemistry results. Diets with higher FB inclusion tended to be slightly less digestible compared with the control. Dogs also defecated more frequently and had a tendency to produce more feces when fed the FB30 diet, but stool quality was not affected by the high FB inclusion. Interestingly, palatability of FB10 and FB30 was lower than the control, but the FB20 did not perform differently from the FB0. This study and previous work by the authors (Corsato Alvarenga and Aldrich, 2019) indicated that processed FB were a safe ingredient to be used in dog foods. However, a longer feeding trial should be conducted to confirm their long-term safety. Further, dehulled FB should not be included at levels above 20% to maintain palatability and stool quality.

## Abbreviations

AOAC: Association of Official Analytical Chemists
ATTD: apparent total tract digestibility
CBC: complete blood count
DM: dry matter
EDTA: ethylenediaminetetraacetic acid
FB: faba beans
FB0: rice-based diet with no FB
FB10: diet with 10% FB
FB20: diet with 20% FB
FB30: diet with 30% FB
G6PD: glucose-6-phosphate dehydrogenase
LOQ: limit of quantification
ME: metabolizable energy
OM: organic matter
RBC: red blood cell
TDF: total dietary fiber
TIU: trypsin inhibitor units
